# Sample size determination for external pilot cluster randomised trials with binary feasibility outcomes: a tutorial

**DOI:** 10.1186/s40814-023-01384-1

**Published:** 2023-09-19

**Authors:** K. Hemming, M. Taljaard, E. Gkini, J. Bishop

**Affiliations:** 1 Institute of Applied Health Research, University of Birmingham, Birmingham, UK; 2 Clinical Epidemiology Program, Ottawa Hospital Research Institute, Ottawa, ON Canada; 3 School of Epidemiology and Public Health, University of Ottawa, Ottawa, ON Canada

## Abstract

Justifying sample size for a pilot trial is a reporting requirement, but few pilot trials report a clear rationale for their chosen sample size. Unlike full-scale trials, pilot trials should not be designed to test effectiveness, and so, conventional sample size justification approaches do not apply. Rather, pilot trials typically specify a range of primary and secondary feasibility objectives. Often, these objectives relate to estimation of parameters that inform the sample size justification for the full-scale trial, many of which are binary. These binary outcomes are referred to as “feasibility outcomes” and include expected prevalence of the primary trial outcome, primary outcome availability, or recruitment or retention proportions.

For pilot cluster trials, sample size calculations depend on the number of clusters, the cluster sizes, the anticipated intra-cluster correlation coefficient for the feasibility outcome and the anticipated proportion for that outcome. Of key importance is the intra-cluster correlation coefficient for the feasibility outcome. It has been suggested that correlations for feasibility outcomes are larger than for clinical outcomes measuring effectiveness. Yet, there is a dearth of information on realised values for these correlations.

In this tutorial, we demonstrate how to justify sample size in external pilot cluster trials where the objective is to estimate a binary feasibility outcome. We provide sample size calculation formulae for a variety of scenarios, make available an R Shiny app for implementation, and compile a report of intra-cluster correlations for feasibility outcomes from a convenience sample. We demonstrate that unless correlations are very low, external pilot cluster trials can be made more efficient by including more clusters and fewer observations per cluster.

**Supplementary Information:**

The online version contains supplementary material available at 10.1186/s40814-023-01384-1.

## Key messages

- Justifying sample size for an external pilot trial is a reporting requirement, but few pilot cluster trials report a clear rationale for their chosen sample size.
- In this tutorial, we demonstrate how to justify sample size in external pilot cluster trials where the objective is to estimate a binary feasibility outcome.
- We make available an R Shiny app for implementation and compile a report of intra-cluster correlations for feasibility outcomes from a convenience sample.

## Introduction

### What are pilot cluster trials and why are they needed?

Cluster randomised trials (CRTs) involve randomisation of whole groups of individuals, referred to as clusters, to intervention or control conditions [6, 7]. Cluster randomisation has become an increasingly popular design choice over the past couple of decades [18, 19]. As with any randomised trial, cluster trials require careful feasibility testing and piloting of trial processes before initiating a full-scale trial, especially given its added complexity, financial cost and resources. Thus, it can be good practice to conduct either a feasibility study (for example to identify if the intervention is acceptable) or a pilot trial (to pilot a future study) in advance of the full-scale cluster trial [12]. Unlike full-scale trials, external pilot trials should not formulate primary objectives around testing effectiveness but, instead, focus on determining feasibility of the full-scale trial. The focus of pilot work is often around estimating the parameters that will be used in a sample size calculation of the full-scale trial, including estimating recruitment, retention and primary outcome availability [5]. Thus, pilot trials therefore typically specify a range of primary and secondary feasibility objectives, which often include estimation of binary feasibility outcomes (e.g. proportion with primary outcome availability).

### Why sample size justification is necessary in pilot trials?

This different focus of pilot studies means conventional sample size justification methods, which focus on hypothesis testing, do not apply. However, external pilot studies still require careful sample size justification, both so that funders can be satisfied that pilot trials will be of sufficient size to meet their objectives and also to ensure research integrity (it would be unethical to conduct a pilot trial that was either much larger than it needed to be or much smaller than would be useful) [10]. When the key objectives relate to binary feasibility outcomes, the sample size can be justified on the basis of estimating a proportion with “sufficient” or “acceptable” precision. Clearly, the concept of “sufficient” precision is not an objective construct. However, some idea of how precise a particular proportion or percentage can be estimated is important — as it provides insights into how well the pilot trial will be able to meet its objectives. For example, if it were known that a pilot trial will only be able to estimate a key proportion to within 20% error — this would mean at the end of the study, it would be known that this percentage is somewhere between 30 and 70% — which is likely not very informative to design most full-scale trials.

### How to perform sample size calculations for pilot cluster trials?

As with the case of individually randomised trials, sample size justification for pilot trials depends on specification of a series of parameters for which there is likely to be much uncertainty. For example, if estimating the prevalence of a key outcome, then an estimate of this prevalence is needed to input into these calculations. These calculations are further complicated in cluster randomised trials due to the need to account for the intra-cluster correlation coefficient. Whilst estimates of intra-cluster correlations for effectiveness outcomes are available within the literature [1, 3, 21], this is not the case for intra-cluster correlation coefficients for feasibility outcomes. Thus, whilst it is anticipated that correlations for feasibility outcomes are higher than those for effectiveness outcomes, there is little evidence to back this up [10]. This is important because these parameters are key to estimation of the required sample size.

### How are pilot cluster trials justifying their sample size currently?

Unfortunately, few pilot CRTs report a clear rationale or justification for their chosen sample size [5]. Furthermore, most pilot CRTs by their nature are small: typical number of clusters included in pilot CRTs is about 8 [*IQR*: 4 to 16] and average cluster size 32 [*IQR*: 14 to 82]) [5]. Whether or not these sizes are too small to estimate key feasibility outcomes with reasonable precision depends crucially on likely values of intra-cluster correlation coefficients [10]. In addition, there is often a trade-off between increasing the number of clusters versus the size per cluster, especially when wider considerations mean only a limited number of clusters can be included [14]. These considerations complicate sample size determination for pilot CRTs.

## Methods

### Objectives

In this tutorial, our objective is to provide statisticians with accessible guidance on how to determine the sample size (both number of clusters and number per cluster) in external pilot CRTs to estimate a key proportion, such as the proportion with primary outcome available, with a degree of precision that will mean that something useful can be inferred about this proportion at the end of the pilot trial. To this end, we provide examples of margins of errors, so that researchers can understand the implications of conducting pilot trials that do not allow useful inferences about target parameters. In particular we compile a completion of intra-cluster correlation coefficients for feasibility outcomes from a small convenience sample of trials. We provide worked examples, outline formulae, and provide resources and recommendations for setting typical parameters required in these sample size calculations. We make available an R Shiny app to implement these calculations. Finally, in the discussion, we provide reminders of what objectives are typically infeasible within the remit of a pilot trial, such as preliminary estimates of effect sizes and estimates of intra-cluster correlations. Ultimately, this should lead to pilot trials that are designed in such a way so as to be able to meet their objectives.

### Margin of error for estimated proportions in cluster randomised trials

When the key objectives of a pilot cluster trial relate to estimation of a binary feasibility outcome (e.g. proportion with primary outcome availability), the sample size can be justified on the basis of estimating a proportion with sufficient or acceptable precision (see later explanation of “sufficient”). In this case, sample size justification will depend on the number of clusters, the cluster sizes, the anticipated intra-cluster correlation coefficient for the feasibility outcome, and the anticipated proportion for that outcome. Using a similar approach to Eldridge et al. [10], we can determine the margin of error or precision that will be realised around a given proportion under a given sample size. Under the central limit theorem (thus making large sample approximations), the “margin of error” for the estimation of a combined proportion (across both arms) is as follows [8, 10]:

1 $$\text{Margin of Error (MoE)}={t}_{\alpha /2,k-2}\sqrt{\frac{\left(1+\left(m-1\right)\rho \right)\pi (1-\pi )}{mk}}$$

where:

$\alpha$ = Value to specify a 100*(1-$\alpha$)% confidence interval (set at 5% here)

m = The number of observations per cluster

k = The total number of clusters (both arms)

$\pi$ = Best guess of the proportion being estimated

$\rho$ = Intra-cluster correlation coefficient (ICC) for the proportion being estimated

MoE = Margin of error

If proportions are expected to be close to the bounds of 0 or 1, then these approximations might not be appropriate [8]. See supplementary material for an alternative approach, known as the Wilson-Score approach [20]. We use a *t*-distribution with k-2 degrees of freedom as normal approximations are known to not hold in settings with fewer than about 40 clusters (which is typically the case in pilot cluster trials) [5, 17]. In settings where it is known that there might be some variation across clusters in their sizes, Eq. 1 can be modified as follows:

2 $$\text{Margin of error (MoE)}={t}_{\alpha /2,k-2}\sqrt{\frac{\left(1+\left((1+{cv}^{2})\overline{m }-1\right)\rho \right)\pi (1-\pi )}{\overline{m}k} }$$

where $\overline{m }$ is the anticipated average cluster size and $cv$ is the coefficient of variation of cluster sizes [9]. Of note, this provides a conservative estimate of extra inflation due to variation in cluster sizes and can likely be ignored if the anticipated coefficient of variation is less than about 0.23 [9].

These equations allow estimation of precision of a pooled proportion across *k* clusters. In pilot CRTs, *k* will often represent the total number of clusters included. Where it is anticipated, these proportions might vary across arms; they can be estimated separately in each arm, in which case *k* would be the number of clusters in each arm. Pooling across arms naturally increases the sample size and in practice can often be the only feasible approach. Moreover, the intra-cluster correlation coefficient (*ρ*) relates to the primary feasibility outcome (e.g. proportion on whom primary outcome is available). It is important to note that these intra-cluster correlation coefficients might very well be different to those that relate to the anticipated primary effectiveness outcomes (see later section for guidance on likely values).

These margins of errors are a measure of precision that are anticipated to be realised, under various assumptions about key parameters, and are probably best understood by considering likely ranges of 95% confidence intervals for different proportions and different margins of errors (Table 1). For example, if the percentage of participants on whom the primary outcome is available is anticipated to be about 50%, setting the margin of error to 5% would result in the 95% confidence interval ranging from 45 to 55% (assuming the realised percentage was also 50%). This 95% confidence interval can be used as an aid to communicating how precisely the proportion is estimated. This margin of error is sometimes referred to as the half-width of the confidence interval. Increasing the margin of error increases the width of the confidence interval. For example, a margin of error of 10% around an anticipated percentage of 50% would result in a 95% confidence interval ranging from 40 and 60% — that is, much less precise.

**Table 1:** Aid to communicate meaning of margin of error

		95% CI for different margins of error (as percentage)			
		**1%**	**5%**	**10%**	**20%**
Anticipated proportion (presented as a percentage for ease of understanding)	**5%**	4–6%	0–10%	NA	NA
	**15%**	14–16%	10–20%	5–25%	NA
	**25%**	24–26%	20–30%	15–35%	NA
	**50%**	49–51%	45–55%	40–60%	30–70%

*NA* confidence interval not presented as would be out of boundary of parameter space (< 0 or > 100); *CI* confidence interval

To determine the margin of error around a given proportion estimated from a pilot CRT, various parameters must be specified, including the anticipated value for the proportion to be estimated ($\pi$). This can appear counter-intuitive but is nonetheless necessary. These prior estimates can often be informed by similar studies or other literature. Furthermore, for cases where these proportions are not anticipated to be very low or very high, we show below how assuming that the proportion is 50% can be a sensible approach.

### Conducting sample size calculations for pilot CRTs

#### Scenario 1: Determining the maximum likely error given a fixed number of clusters and fixed cluster size

To achieve the largest possible margin of error for a given sample size, and thus set a conservative upper bound on the required sample size, a proportion of *π* = 0.5 (i.e. assuming that the anticipated percentage of 50%) can be used in calculations. This is the most conservative approach because at proportions of 0.5, the standard error π (1-π) is at its largest. In these settings, the margin of error can be thought of as the “maximum likely error”:

3 $$\text{Maximum likely error (max. error)}={t}_{\alpha /2,k-2}\sqrt{\frac{\left(\left(1+\left(m-1\right)\rho \right)\right)\times 0.25}{mk}}$$

**Fig. 1:**
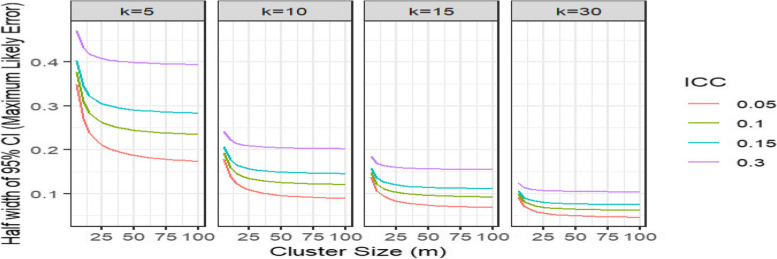
Maximum likely error by cluster size (m), number of clusters (k) and intra-cluster correlation coefficient (ICC)

Figure 1 illustrates how the maximum likely error decreases with increasing number of clusters. The figure also illustrates how the maximum likely error decreases with increasing cluster size, but as in full-scale cluster trials, there is a plateauing effect such that beyond a certain point the material contribution of observations to statistical precision will become negligible [14]. Furthermore, the maximum likely error is also highly dependent on the anticipated intra-cluster correlation coefficient. The plateauing effect can be seen to kick in at smaller cluster sizes when the intra-cluster correlation coefficient is larger — meaning that the incremental contribution of increasing cluster sizes become more questionable in such settings. Perhaps most strikingly, this plot illustrates how for pilot CRTs with 10 or fewer clusters the maximum likely error is likely to be large. For example, for an intra-cluster correlation coefficient of 0.05, with 10 clusters and cluster size of 25 (the size of a typical pilot CRT [5]), the maximum likely error is around 10% (meaning a percentage of 50% would be estimated to be between 40 and 60%),for an intra-cluster correlation coefficient of 0.10, this increases to be between 30 and 70%.

#### Scenario 2: Determining the number of clusters for fixed maximum likely error when cluster sizes are fixed

The formulae above can be used to determine the maximum likely error when the number of clusters and cluster sizes are fixed, for example, when the trial only has resources to include a fixed number of clusters and when there are a fixed number of participants available within each cluster. Alternatively, the cluster size may be fixed and it may be of interest to determine the number of clusters needed for a specified maximum likely error.

We rearrange the above equations to obtain the minimum number of clusters (*k*) and to achieve a desired margin of error (MoE) around an anticipated proportion ($\pi$), for a fixed cluster size (*m*), and assumed intra-cluster correlation coefficient ($\rho$):

4 $$\begin{array}{c}{\text{MoE}}>{t}_{\alpha /2,k-2}\sqrt{\frac{\left(1+\left(m-1\right)\rho \right)\pi \left(1-\pi \right)}{mk}}\\ k{\left(\frac{MoE}{{t}_{\alpha /2,k-2}}\right)}^{2}>\frac{\left(1+\left(m-1\right)\rho \right)\pi (1-\pi )}{m}\\ k>\frac{\left(1+\left(m-1\right)\rho \right)\pi \left(1-\pi \right)}{m{\left(\frac{MoE}{{t}_{\alpha /2,k-2}}\right)}^{2}}\end{array}$$

To be conservative, and setting $\pi$ = 0.5 to provide a specified maximum likely error (*max error*), this simplifies to the following:

5 $$k>\frac{1+\left(m-1\right)\rho }{4m{\left(\frac{Max\ Error}{{t}_{\alpha /2,k-2}}\right)}^{2}}$$

In practice, the value for *k* needs to be a whole number and so needs to be rounded up. Furthermore, in a randomised pilot trial (with a 1:1 allocation), this would also need to be rounded up to the nearest even number. Again, this formula can be modifed for varying cluster sizes:

$$k>\frac{1+\left((1+{cv}^{2})\overline{m }-1\right)\rho }{4m{\left(\frac{Max\ Error}{{t}_{\alpha /2,k-2}}\right)}^{2}}$$

#### Scenario 3: Determining the cluster size for a specified maximum likely error and fixed number of clusters

Alternatively, it might be the case that the number of clusters is fixed, the desired margin of error fixed, and it is required to determine the cluster size. To determine the number of observations per cluster (*m*) that are needed for a fixed number of clusters (*k*) and intra-cluster correlation coefficient ($\rho$) to estimate a proportion with a margin of error of MoE, we rearrange the above equation to obtain:

$${\text{MoE}}>{t}_{\alpha /2,k-2}\sqrt{\frac{\left(1+\left(m-1\right)\rho \right)\pi (1-\pi )}{mk}}$$

$$m{\left(\frac{MoE}{{t}_{\alpha /2,k-2}}\right)}^{2}>\frac{\left(1+\left(m-1\right)\rho \right)\pi (1-\pi )}{k}$$

$$\frac{m}{1+\left(m-1\right)\rho }>\frac{\pi (1-\pi )}{k{\left(\frac{MoE}{{t}_{\alpha /2,k-2}}\right)}^{2}}$$

This can be rearranged easily, to solve for *m*. Again, this simplifies, when assuming $\pi$ = 0.5, to provide a specified maximum likely error *(max error)*:

6 $$m>\frac{1-\rho }{4k{\left(\frac{Max\ Error}{{t}_{\alpha /2,k-2}}\right)}^{2}-\rho }$$

Modify these for varying cluster sizes:

$$m>\frac{1-\rho }{4k{\left(\frac{Max\ Error}{{t}_{\alpha /2,k-2}}\right)}^{2}-(1+{cv}^{2})\rho }$$

### R Shiny app for sample size justification for binary feasibility outcomes in cluster randomised trials

An R Shiny app that implements these calculations can be found at https://clusterrcts.shinyapps.io/RshinyAppPilotStudies/. To use the app, users need to specify the intra-cluster correlation coefficient for the outcome of interest, the number of clusters, the average cluster size along with a coefficient of variation of cluster sizes, the anticipated proportion to be estimated (with a default of 0.5 to be conservative), and the required confidence limit (with a default of 95%). The resulting output includes a graphical representation of the anticipated half-with of the confidence interval (or equivalently the maximum likely error) against the number of clusters (default range 0 to 100 clusters), text interpreting the output (at the number of clusters fixed by the user), and tabulated output (for exact values where required). Where proportions are expected to be very low or high (say less than 10% or greater than 90%), then the Wilson-Score version of the estimates should be used (supplementary material A).

### Specification of intra-cluster correlation coefficients

Designing a cluster pilot trial using the methodology above requires an advance estimate for the intra-cluster correlation coefficient (for the feasibility outcome). One way of obtaining an estimate is to use values reported in previously published trials. Unfortunately, reporting correlations for feasibility outcomes is not standard practice [11]. Another option is to use rules of thumb. In full-scale cluster trials, typical intra-cluster correlation coefficients tend to be quite small (e.g. in primary care datasets, the upper inter-quartile range for the intra-cluster correlation coefficient is 0.02) [1, 3]. However, it is known that intra-cluster correlation coefficients for process measures (variables that capture the process of patient care, such as adherence to clinical guidelines) tend to be larger than for clinical outcomes [3]. This is because process outcomes are more likely to vary by centre due to staff behaviour. For example, across a large sample of process outcomes measuring maternity care, the upper interquartile range for the intra-cluster correlation coefficient is 0.33 [21]. For similar reasons it has been hypothesised that intra-cluster correlation coefficients for feasibility outcomes (variables that measure trial processes, such as primary outcome availability) might also be larger than for clinical outcomes [10]. Therefore, whilst when designing full-scale CRTs, it is typical to assume a fairly low intra-cluster correlation coefficient,when designing pilot trials, intra-cluster correlation coefficients are anticipated to be larger [10].

Table 2 reports empirical estimates of intra-cluster correlation coefficients for feasibility outcomes from a convenience sample of randomised trials. These include cluster randomised trials and multicentre individually randomised trials and both pilot and full-scale trials (see supplementary material B for details of studies included). Note that data from both cluster randomised trials and individually randomised multicentre trials allow estimation of correlations within clusters/centres. All outcomes relate to what might be considered feasibility outcomes, including primary and secondary outcome availability, measures of adherence, and questionnaire return. All are UK- or European-based trials; most include more than 20 clusters/centres, and the total sample size is greater than 500 for the majority. We estimate the correlations on the proportions scale (as is appropriate for binary outcomes) [24, 25], using REML and implemented in Stata 17 using *mixed* (with the exception for two outcomes where *mixed* failed to converge and we used ANOVA implemented in Stata using *loneway*). The upper interquartile range across all available intra-cluster correlation coefficients is 0.11 [*IQR*: 0.05 to 0.18].

**Table 2:** Estimates of intra-cluster correlation coefficients for feasibility outcomes from a convenience sample of trials

Trial	Feasibility outcome description	Design	Setting	No. of clusters	TSS	Proportion	ICC	SE	LCI	UCI
KG SEAR	Agree to be randomised out of those eligible	RCT	Secondary care UK	19	760	0.34	0.08	0.04	0.03	0.20
INTERVAL	Availability of primary outcome: bleeding gums	RCT	Dentistry UK	51	2374	0.68	0.09	0.02	0.05	0.14
	Availability of secondary outcome: patient-reported outcome	RCT	Dentistry UK	51	2374	0.72	0.11	0.03	0.07	0.18
IQuaD	Availability of primary outcome: bleeding gums	CRT	Dentistry UK	63	2021	0.66	0.07	0.02	0.04	0.12
	Availability of secondary outcome: patient self-efficacy	CRT	Dentistry UK	63	2021	0.72	0.05	0.01	0.03	0.09
VUE	Availability of outcome: patient-reported prolapse symptoms (POP-SS)	RCT	Women’s Health UK	46	781	0.82	0.01	0.01	0.00	0.15
	Availability of outcome: quality of life^a^	RCT	Women’s Health UK	46	781	0.83	0.00	0.01	0.00	0.03
Trigger	Adherence to transfusion policy	CRT	UK Transplant	6	935	0.70	0.12	0.07	0.04	0.33
	Received at least 1 RBC transfusion	CRT	UK Transplant	6	935	0.41	0.04	0.03	0.01	0.16
Will	Availability of primary outcome 1: severe composite maternal morbidity	RCT	Maternity care UK	45	403	0.96	0.14	0.06	0.06	0.28
	Availability of co-primary outcome: neonatal NICU admission	RCT	Maternity care UK	45	403	0.96	0.23	0.07	0.12	0.39
ROCSS	Availability of primary outcome: hernia within 2 years	RCT	Surgery Europe	37	390	0.82	0.03	0.02	0.01	0.11
ABA-feed	Availability of primary outcome which is breastfeeding at 8 weeks^a^	RCT	Maternity care UK	102	357	0.92	0.00	0.05	0.00	0.9
APPEAL 2	Questionnaire return	CRT (pilot)	Maternity care UK	17	997	0.18	0.04	0.02	0.01	0.11
GBS2	Receipt of antibiotics	CRT	Maternity care UK	20	1622	0.39	0.06	0.02	0.03	0.13
WAVES	BMI availability (baseline)	CRT	Schools UK	54	1467	0.95	0.01	0.01	0.00	0.08
	BMI availability (first follow-up)	CRT	Schools UK	54	1467	0.85	0.12	0.03	0.07	**0.19**
	BMI availability (second follow-up)	CRT	Schools UK	54	1467	0.78	0.08	0.02	0.04	0.13
						**Median**	**0.07**	**0.02**	**0.03**	**0.14**
						**UQR**	**0.11**	**0.04**	**0.05**	**0.18**

*TSS* total sample size, *no. of clusters* number of clusters/centres, *SE* standard error (of ICC), *ICC* intra-cluster correlation coefficient for the feasibility outcome, *LCI* lower 95% confidence interval, *UCI* upper 95% confidence interval, *UQR* upper quartile

^a^Use loneway and reported ICC truncated at zero. Information on trial sources is available in the supplementary material B

As with any sample size justification, it is good practice to explore sensitivity to values assumed, and where appropriate, to assume values for correlations that provide more conservative estimates of maximum likely error, so as to avoid confidence intervals around primary feasibility parameters being wide and uninformative.

## Results

The Antenatal Preventative Pelvic floor Exercises And Localisation (APPEAL) study is a pilot CRT [2]. The unit of randomisation is a community midwifery team with clusters allocated with a 1:1 allocation ratio to intervention or control. Key quantitative objectives relate to determining the proportion of participants with available data for the primary outcome. This information could be used to both infer generalisability and inform future sample size calculations for the full-scale trial.

Here, we illustrate sample size justification related to the key objective of estimating the proportion for whom primary outcome is available. Note that some of these examples match examples in Eldridge [10],slight differences between values presented are due to use of the *t*-distribution (here) as opposed to the z-distribution in Eldridge [10].

Specifically, the objective was to estimate this proportion along with a 95% confidence interval to reflect the uncertainty around this estimate. The anticipated proportion was approximately 0.5, which also corresponds to the proportion yielding the most conservative estimate of precision. For the purposes of the pilot, a fixed random sample was taken from each cluster, and therefore, no allowance for varying cluster sizes is required. Using historical data in a similar setting and for similar clusters, a conservative estimate for the intra-cluster correlation coefficient (for the proportion with primary outcome availability) was estimated to be in the region of 0.10 [2].

### Scenario 1: Estimate the maximum likely error around the primary feasibility outcome given a fixed sample size

It was anticipated that within the pilot trial, it might be feasible to include around 14 clusters each with a sample size of 100 per cluster (i.e. total sample size of 1400). Using Eq. 3, and assuming an intra-cluster correlation coefficient of 0.10 and an anticipated percentage of 50% (percentage for whom primary outcome is available), we calculated that a trial of this size would estimate this primary feasibility outcome with a 95% confidence interval from 40 to 60% or alternatively, to within a maximum likely error of 10% (the half-width of the 95% confidence interval).

### Scenario 2: Estimate the number of clusters needed for a fixed cluster size and specified maximum likely error

Suppose the cluster size is fixed and it is desirable to estimate the percentage with primary outcome availability to within ± 10%. For a fixed cluster size, the number of clusters needed to obtain a maximum likely error of 10% is presented in Table 3 (using Eq. 4). For example, with an anticipated cluster size of 100 and setting the maximum likely error to 10%, then for an intra-cluster correlation coefficient of 0.10, 13 clusters are required. After rounding, 14 clusters in total would allow a 1:1 allocation across arms. Note, if the intra-cluster correlation coefficient for this outcome was larger than 0.10, a larger number of clusters would be required. For example, if the intra-cluster correlation coefficient is 0.30, then 32 clusters are required — more than double that which are required under the smaller intra-cluster correlation coefficient of 0.10.

**Table 3:** Illustrative example to determine the number of clusters (k) for a fixed cluster size (m) and set maximum likely error (10%) across range of intra-cluster correlation coefficients (ICCs)

	Number of clusters (k) needed by cluster size (m) and ICC				
	***m* = 10**	***m* = 20**	***m* = 50**	***m* = 75**	***m* = 100**
**0.30**	38	35	33	33	32
**0.15**	26	21	19	18	18
**0.10**	21	17	14	14	13
**0.05**	17	12	10	9	9

Values rounded up to nearest whole number (further rounding up to an even number required if a 1:1 randomised allocation is used).

### Scenario 3: Estimate the cluster size needed for a fixed number of clusters and specified maximum likely error

For a fixed number of clusters, the cluster sizes needed to obtain a maximum likely error of 10% are presented in Table 4 (using Eq. 6). Assuming the anticipated number of clusters is 14 and maximum likely error is 10%, for an intra-cluster correlation coefficient of 0.10, the required cluster size is 51. However, notice that if the intra-cluster correlation coefficient was, for example 0.15, irrespective of cluster sizes, it is not possible to obtain a maximum likely error of 10%. This illustrates the likely futility of setting the cluster sizes as large as 100 in this example (with 14 clusters).

**Table 4:** Illustrative example to determine the cluster size (m) for a fixed number of clusters (k) and set maximum likely error (10%) across a range of intra-cluster correlation coefficients (ICCs)

		Cluster size (m) needed by number of clusters (k) and ICC				
		***k* = 6**	***k* = 8**	***k* = 10**	***k* = 14**	***k* = 20**
**ICC**	**0.30**	NA	NA	NA	NA	NA
	**0.15**	NA	NA	NA	NA	28
	**0.10**	NA	NA	NA	51	12
	**0.05**	NA	276	38	14	8

*NA* non-applicable, meaning that it is not possible to estimate with 10% maximum likely error under that scenario; values rounded up to nearest integer

## Discussion

Justification of sample sizes for external pilot cluster trials is known to be poor [5]. Whilst pilot trials can have several objectives, typical objectives are often related to estimation of prevalence of a binary outcome — for example the proportion completing follow-up. This tutorial guides researchers through sample size justification in these settings, where sample size justification is essentially about determining the number of clusters and cluster size that will allow estimation of these quantities with reasonable precision. We provide these formulae for a variety of scenarios — so as to match variation in practice — for example allowing determination of the number of clusters (where other quantities are fixed) or determining maximum likely error (where the total sample size is fixed). Crucial to this is the pre-specification of “best guesses” of both the anticipated proportion and the intra-cluster correlation coefficient of this outcome. Here, we provide some practical guidance: setting the proportion to be 0.5 in calculations will be the most conservative, and we report a small compilation of estimates of intra-cluster correlation coefficients for feasibility outcomes. An R Shiny app allows implementation of these calculations without requiring the use of statistical packages or coding of these formulae (which can be prone to error).

### Limitations

The methods of calculation used here do have some limitations. Whilst we used the *t*-distribution to accommodate a small number of clusters, we relied on large sample approximations to the variance of a binary outcome. Alternative approaches will be necessary when working on the boundary of the parameter space (proportions close to 0 or 1) [7, 19]. These alternative approaches make fewer assumptions, are not symmetrical and so do not produce confidence intervals outside the boundary of the parameter space, have better coverage properties (at least in the non-clustered setting), and typically require larger sample sizes than under the approach used here [7, 19]. These alternatives are programmed in the accompanying app, but their performance has not been evaluated in the clustered data setting.

We have not considered the method of analysis here. In practice, rather than analysing cluster-level proportions, analysis could be conducted on the logit scale prior to back-transformation. This would mitigate the problem of values out of the bounds of parameter space in the analysis [13]. Analysis of cluster-level proportions, as with sample size justification, should also use *t*-distributions on k-2 degrees of freedom and might need to consider weighting if cluster sizes vary considerably [15]. The use of mixed models and generalised estimating equations should probably be used with some caution because of small sample issues and, if used, should ideally use the *t*-distribution with k-2 degrees of freedom (known as the between-within correction in mixed models) [16].

Furthermore, the methods presented here are based on estimating the proportion across both arms of the study combined (pooled proportion) — and not in each arm separately. Estimation within one arm only is feasible and could be implemented using the calculations presented here by modifying k to be the number in each arm (rather than the number in both arms combined).

### Broader considerations

There are of course wider issues to consider when planning pilot CRTs. Some of these relate to broader aspects of the design, including the selection of clusters and the specification of progression criteria. These might not seem to be directly related to estimation of key proportions, but do have important and related considerations. For example, if the selected clusters are not representative of those to be included in the full-scale trial, the estimated parameters might not be transferable. Moreover, if the progression criteria fix hard and fast rules, for example that follow-up should be greater than 70%, a focus on point estimates might be misguided if confidence intervals are wide (although arguably, values at the tails of the confidence intervals might be equally unlikely). We have also only considered external pilot trials. Alternatives are to conduct what is known as an internal pilot trial, in which the main trial continues in a seamless way, provided progression criteria hold. Internal pilots can have important benefits in minimising the required number of clusters and participants, especially when an external pilot trial might be required to be too large to fully align with its objectives [23].

We have not considered sample size calculation for non-binary outcomes, such as estimation of the standard deviation of continuous outcomes, which might be of interest in a pilot trial [23]. Purposively, we have not considered the estimation of the intra-cluster correlation of the intended primary outcome (for use in the definitive sample size calculation) nor the so-called “preliminary” estimates of effect sizes for the intended primary outcome. Others have underscored the importance of not estimating effects of intended primary outcomes in pilot trials — as pilot trials are typically too small to estimate this with any reasonable precision — and this clearly should be left to the full-scale trial [12]. Furthermore, although estimation of the intra-cluster correlation coefficient seems to be a useful objective of a pilot trial, unfortunately in practice, pilot trials typically have too few clusters to yield informative estimates (except in settings of pilot trials with many clusters, or very large clusters, or very low intra-cluster correlations) [10].

### Further contextual issues

The average number of clusters included in pilot cluster randomised trials is just 8 (with an upper quartile range of 16), and the average cluster size is 32 (with an upper quartile range of 82) [5]. It is known that typical pilot CRTs will therefore not be able to estimate key proportions with reasonable precision [10]. The results presented here underscore this important message. For example, if intra-cluster correlation coefficients are in the region of 0.10, typical pilot CRTs will only be able to estimate key proportions to within 20% error. Realistically, this means that the typical pilot CRT, with say 10 clusters and a sample size of about 50 per cluster, might only be able to infer that some key proportion is somewhere between 30 and 70% — which is not very informative. However, this critically depends on the intra-cluster correlation coefficient. Intra-cluster correlation coefficients are highly context dependent, and it might be that in scenarios where correlations are lower, valuable information might be obtainable from pilot CRTs of these sizes. For example, for intra-cluster correlation coefficients in the region of 0.05, it might be possible to estimate these quantities to within 10% maximum likely error — but even this means confidence intervals around key parameters will be wide. Thus, information on intra-cluster correlation coefficients (for pilot study outcomes) is crucial to allow a realistic expectation of the likely information available from pilot CRTs [11]. The CONSORT for cluster statement includes a recommendation that trialists report estimates of ICC for outcomes [4]. We would recommend that authors of full scale and pilot trials report ICCs for feasibility outcomes, if even in the supplementary material, as these can help inform the design of pilot CRTs.

There are substantial risks with running small cluster trials [22], and this also holds unfortunately for pilot cluster trials with a small number of clusters [10]. The tools provided here should allow researchers to appreciate this directly, when they are considering designing their own pilot CRT. Ultimately, this should help to increase awareness that to properly estimate key parameters in pilot trials, the pilot trials do need to be large. Of course, this leads to somewhat of a conundrum: pilot trials by their nature are intended to be small. Potential solutions, where practical, would consist of including more smaller clusters rather than many larger clusters.

### Summary

Sample size in pilot CRTs needs clear justification. When trying to estimate key quantitative parameters, such as primary outcome availability, justification depends on the number of clusters, the cluster sizes, the anticipated intra-cluster correlation coefficients for the pilot outcome, and the anticipated proportion of that outcome. Key to all this is the intra-cluster correlation coefficient. Intra-cluster correlation coefficients for key feasibility outcomes measured in pilot trials might be substantially larger than correlations for typical clinical outcomes. For likely values of these correlations, pilot CRTs need to be larger than most typically sized pilot CRTs to be informative. Unless correlations are very low, pilot cluster trials can be made more efficient by not including very large cluster sizes.

The work presented here would suggest that if pilot cluster trials are to be informative in estimating key binary feasibility outcomes, then they need around 15 clusters (the size of each cluster is less important but should be greater than about 30 per cluster) under a working assumption that the intra-cluster correlation coefficient is in the region of 0.10. This would ensure that the resulting half-width of the confidence interval would be within 10 percentage points (i.e. if the outcome occurred in 50% of cases, this would have a 95% CI from 40 to 60%). Confidence intervals wider than this (e.g. from 30 to 70%) are unlikely to be very informative. Only in settings where there is good evidence to support the intra-cluster correlation being 0.05 or less would around 10 clusters be sufficient.

### Supplementary Information

**Additional file 1: Supplementary material A. **The Wilson-Score method for interval estimation for clustered data.

**Additional file 2. **

## Data Availability

The data are available on request from original studies.
