# Supplementary material for: Sample size determination for external pilot cluster randomised trials with binary feasibility outcomes: a tutorial
Source: Pilot Feasibility Stud. 2023 Sep 19;9:163. doi: 10.1186/s40814-023-01384-1 (PMC10507981; doi:10.1186/s40814-023-01384-1)
Supplement: Supplementary file 1 — Additional file 1: Supplementary material A. The Wilson-Score method for interval estimation for clustered data. [file 40814_2023_1384_MOESM1_ESM.docx]

**Supplementary material A: the Wilson-Score method for interval estimation for clustered data**

In section 3.4.3 Newcombe (2012) outlines the Wilson-Score interval approach for the estimation of confidence intervals for proportions (independent data). It is stated that this approach improves performance when n (total sample size, independent data) is small or p (proportion with outcome) or q (=1-p) are small. This confidence interval is obtained by solving a quadratic equation (equation 3.4 in Newcombe 2012) to give two solutions (one for the upper limit and one for the lower limit). These two solutions are provided at equation 3.5 in Newcombe 2012.

For independent data, z is a standard normal, p the proportion, n the total sample size, r the number of events (=np) then:

The quadratic to be solved is (equation 3.4 in Newcombe 2012):


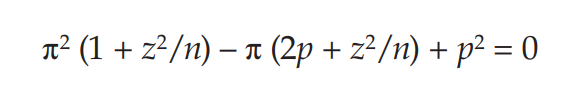


And resulting two solutions are (equation 3.5 in Newcombe 2012):


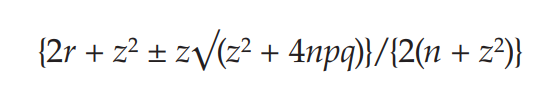


For clustered data, we replace n in the above, with n=mk[1+(m-1)ICC] (i.e., the effective sample size) and r as pmk[1+(m-1)ICC]. Where m is the cluster size, k the number of clusters, and ICC is the intra-cluster correlation coefficient for the proportion being estimated.

**References**

[Newcombe 2012] Newcombe RG. Confidence intervals for proportions and related measures of effect size. CRC press; 2012 Aug 25.
